# Rapid Detection of *Klebsiella pneumoniae* in Infants With Severe Infection Disease by Instant Nanofluorescent Microspheres‐Immunochromatography Combined With Recombinase Polymerase Isothermal Amplification Technology

**DOI:** 10.1002/jcla.70348

**Published:** 2026-10-08

**Authors:** Yiping Liu, Yi Lu, Xin Wang, Hanzhong Yu, Yumeng Lu, Minghui Chen, Feifan Zhu, Xing Lv, Li Wang, Ying Chen

**Affiliations:** ^1^ School of Medical Technology Xuzhou Medical University Xuzhou China; ^2^ School of Chemical Engineering and Technology China University of Mining and Technology Xuzhou China; ^3^ Department of Clinical Laboratory Xuzhou First People's Hospital Xuzhou China; ^4^ School of Medical Imaging Xuzhou Medical University Xuzhou China

**Keywords:** infants, *Klebsiella pneumoniae*, POCT, rapid detection, RPA‐FICTS technology

## Abstract

**Background:**

*Klebsiella pneumoniae* (KP) is an important and virulent pathogen causing acute respiratory infections in infants. Currently, traditional methods for detecting *Klebsiella pneumoniae* in babies are labour‐intensive and time‐consuming, and their accuracy and sensitivity require improvement. Recombinant enzyme polymerase amplification technology (RPA) has the advantages of good specificity, high sensitivity, and isothermal amplification. It can be combined with fluorescence immunochromatography test strip (FICTS) based on nanofluorescent microspheres (nFM) to achieve rapid, low‐cost, high sensitivity, and specific detection of *Klebsiella pneumoniae* in infants.

**Methods:**

*mdh* was selected as a genus‐specific gene from *Klebsiella pneumoniae* , and recombinant polymerase isothermal amplification—nanofluorescence microsphere immunochromatography (RPA‐FICTS) was established by using standard strains. The sensitivity and specificity of this detection technology can be improved by the magnetic bead purification step of RPA nucleic acid amplification products. Subsequently, rapid quantitative detection of *K. pneumoniae* in infant samples was performed using fluorescent immunochromatographic test strips. A total of 19 clinical samples of *Klebsiella pneumoniae* (KP) and 7 clinical samples of non‐ *Klebsiella pneumoniae* (nKP) were detected by the new detection technology platform established in this paper, and the specificity and sensitivity of RPA‐FICTS were verified by agarose gel electrophoresis.

**Results:**

Under the optimal detection conditions, the maximum detection limits of RPA‐GE (agarose gel electrophoresis) and RPA‐FICTS are 9.03 × 10^−3^ and 9.03 × 10^−8^ ng/μL. RPA‐FICTS can quickly detect *Klebsiella pneumoniae* and specifically distinguish between *Klebsiella pneumoniae* and non‐*Klebsiella pneumoniae*. Compared with traditional detection methods, this method has a shorter detection time (55 min), strong specificity, high sensitivity, and simple operation, providing a new method for rapid detection of human *Klebsiella pneumoniae* .

## Graphical abstract

Schematic illustration of the RPA‐FICTS platform for rapid detection of *Klebsiella pneumoniae* in infants. Genomic DNA extracted from clinical samples is amplified by recombinase polymerase amplification (RPA) targeting the mdh gene at 37°C, and the biotin‐ and digoxigenin‐labeled amplicons are subsequently detected on a nanofluorescent microsphere‐based fluorescence immunochromatographic test strip (FICTS), enabling portable quantitative fluorescence readout within 55 min with high sensitivity and specificity.

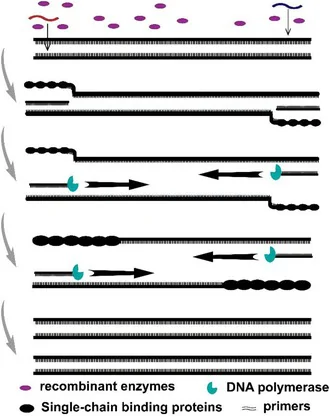

## 1 Introduction

*Klebsiella pneumoniae* belongs to the enterobacterales and is a common pathogenic bacterium in hospital infections. It can cause infections in multiple parts of the body, including the lungs, liver, urethra, blood, and brain. In severe cases, it can cause severe disseminated infections and pose significant harm to humans [1]. Clinical studies show that drug‐resistant *Klebsiella pneumoniae* is highly contagious in infants, and infections caused by *Klebsiella pneumoniae* can lead to sepsis in newborns, often with high mortality [2, 3], resulting in poor patient prognosis and posing key clinical challenges for anti‐infective therapy [4]. As a result, the prevention and control of various common pathogenic microorganisms in daily life is particularly important, and accurate, fast, and real‐time detection technology for pathogenic microorganisms is a necessary means of preventing and controlling pathogenic microorganisms [5]. Traditional microbial detection methods require the cultivation of a large number of bacteria, selective cultivation of the required bacteria, purification, biochemical reaction identification, and other steps. These methods are cumbersome, time‐consuming, and labor‐intensive, and thus cannot meet the needs of rapid, real‐time detection and prevention of pathogenic microorganisms [6]. The most commonly used detection methods in laboratories, such as PCR detection, require expensive PCR amplification equipment to assist in completing the detection and have disadvantages such as long time consumption and cumbersome operation, which are not conducive to clinical testing [7]. Recombinase polymerase amplification (RPA) technology was invented by scientists Piepenburg et al. in Cambridge, UK in 2006 [8] and produced by TwistDx Biotechnology in 2014 as a commercial detection kit. The advantages are short reaction time, high specificity, high sensitivity and simple instrumentation [9, 10]. The principle is shown in Figure 1. In the presence of ATP, the recombinant enzyme uvsX binds to the primer under the action of uvsY to form a complex. The compound recognizes the target sequence of double‐stranded DNA and can open the double strand to form a D‐loop structure. At this time, the gp32 single‐stranded protein will bind to the single stranded DNA to prevent it from forming a double‐stranded structure again. Subsequently, the uvsX protein separates from the primer, and the primer extends from the 3′ end to amplify the DNA, ultimately forming a new DNA strand. The reaction can be performed at a constant temperature of 37°C–42°C and completed within 30 min [11].

**FIGURE 1:**
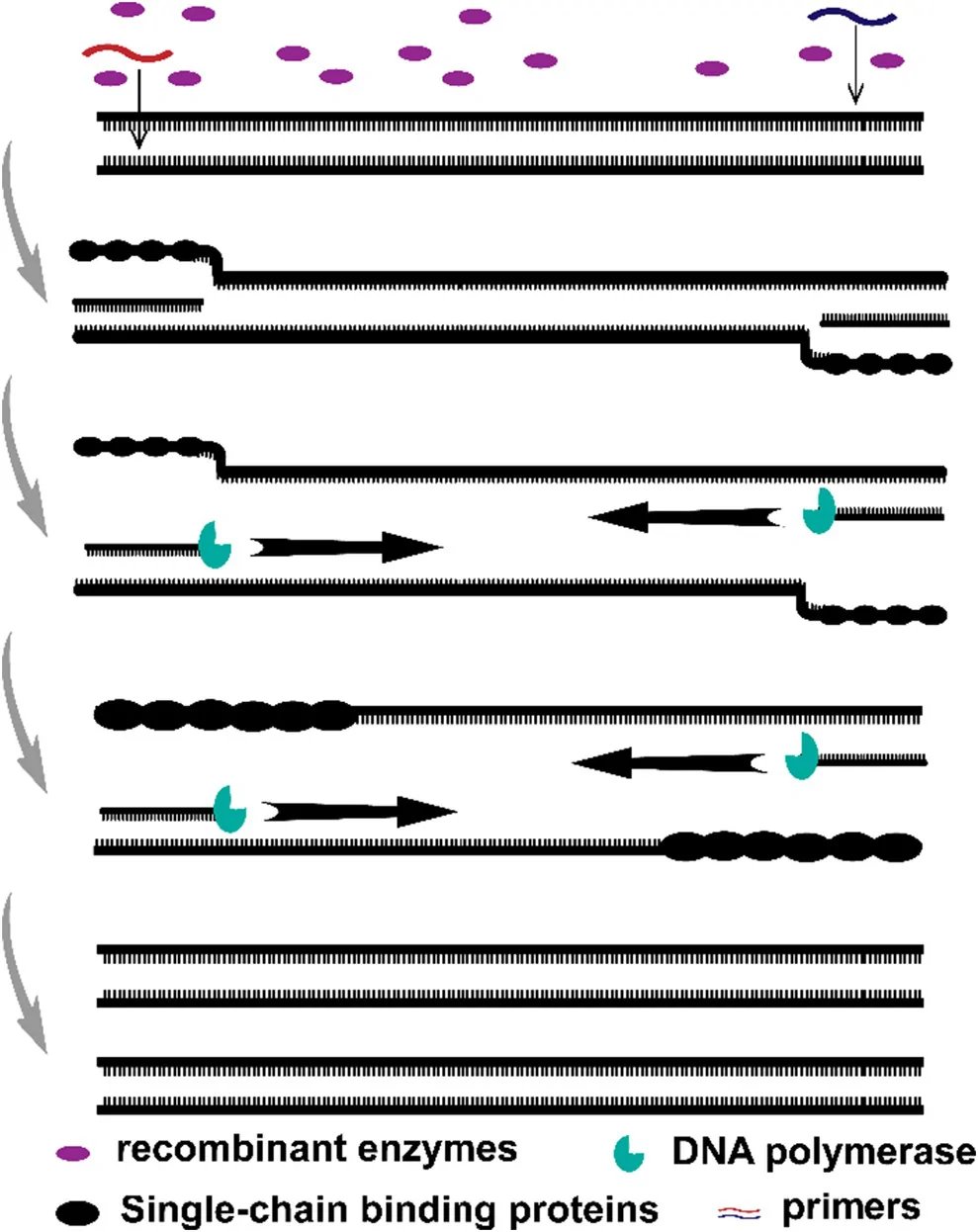
Diagram of RPA principles.

Immunochromatographic analysis (ICA) began in the late 20th century and is a rapid detection technology that combines chromatography and immune technology [12]. The advantage of this detection process is its simplicity and low cost [13]. It has broad application prospects and has been widely used in animal drug residue detection, environmental monitoring, disease diagnosis, food safety, hair sample morphine detection, and other aspects [14, 15]. With the development of nanofluorescent microspheres and nanomagnetic bead technology, new materials with excellent magnetic properties have emerged. These properties include superparamagnetism, quantum size effects, surface effects, and macroscopic quantum tunneling effects [16]. Such materials enable quantitative detection by measuring fluorescence intensity [17] and have been applied in medical testing and other fields [18].

In this assay, we successfully established a method combining nanofluorescent microsphere‐based immunochromatography with recombinase polymerase amplification (RPA‐FICTS) for the isothermal and quantitative detection of *K. pneumoniae* in infants with severe infectious diseases. This method has the potential to serve as an efficient point‐of‐care testing (POCT) technology that does not require expensive instruments, making it suitable for on‐site detection of infectious illnesses such as neonatal pneumonia, septicemia, and bronchopneumonia. Meanwhile, the nanofluorescent microspheres we used are coated with streptavidin, which has an extraordinarily high affinity for interacting with biotin [19]. The specific generation of fluorescence can effectively reduce background signals caused by excess primers, enhance specificity, and have higher fluorescence intensity, sensitivity, and selectivity [20].

## 2 Materials and Methods

### 2.1 Bacterial Culture and DNA Extraction

The strain used in this study was a standard *Klebsiella pneumoniae* strain (ATCC 700603), 19 clinical *Klebsiella pneumoniae* strains, and 7 clinical non‐*Klebsiella pneumoniae* strains from the Affiliated Hospital of Xuzhou Medical University and Xuzhou First People's Hospital (Table 1). Strain identification was carried out using traditional bacterial culture methods combined with Vitek 2 Compact (BioMerieux, France); all DNA samples of pathogenic strains were then stored in a refrigerator at −80°C. The DNA extraction samples were performed using the TIANGEN Bacterial DNA Kit (TIANGEN, Beijing, China) according to the instructions. A260/A280 ratio was measured using a UV spectrophotometer (Meriton SMA 1000, Beijing), and DNA integrity was detected using agarose electrophoresis to evaluate DNA quality. Store the genomic DNA (gDNA) of the above strains in a refrigerator at −20°C until thawed before proceeding with analysis and determination.

**TABLE 1:** The pathogenic bacterial strains of infants used in assays.

No.	Sample number	Age (months)	Sample source	Disease	Species/strain
1	A13	2 days	Sputum	Neonatalpneumonia	*K. pneumoniae*
2	A19	7 days	Sputum	Neonatalpneumonia	*K. pneumoniae*
3	A20	24 days	Sputum	Sepsis	*K. pneumoniae*
4	A22	1	Sputum	Septicopyemia	*K. pneumoniae*
5	A23	1	Sputum	Pneumonia; Sepsis	*K. pneumoniae*
6	A25	1	Sputum	Neonatalpneumonia	*K. pneumoniae*
7	A28	1	Sputum	Bronchitis; Sepsis	*K. pneumoniae*
8	A34	2	Sputum	Bronchopneumonia	*K. pneumoniae*
9	A35	2	Sputum	Severe pneumonia	*K. pneumoniae*
10	A36	2	Sputum	Bronchopneumonia	*K. pneumoniae*
11	A40	2	Sputum	Bronchopneumonia	*K. pneumoniae*
12	A49	2	Sputum	Septicopyemia; Severe pneumonia	*K. pneumoniae*
13	A57	2	Sputum	Bronchopneumonia	*K. pneumoniae*
14	A59	3	Sputum	Ventricular septal defect	*K. pneumoniae*
15	A61	4	Sputum	Bronchopneumonia	*K. pneumoniae*
16	A63	4	Sputum	Necrotizing enteritis	*K. pneumoniae*
17	A65	5	Sputum	Bronchitis	*K. pneumoniae*
18	A66	5	Sputum	Bronchopneumonia	*K. pneumoniae*
19	A125	6	Sputum	Bronchopneumonia	*K. pneumoniae*

### 2.2 Reagents, Chemicals and FICTS Construction

The TwistAmp DNA Amplification Basic Kit used in this study was purchased from TwistDX Company. Other reagents, including ddH_2_O, agar powder, and bacterial culture medium, were obtained from Sangon Biotech (Shanghai) Co. Ltd. The FICTS test strip consists of six components: a sample pad, a nitrocellulose (NC) membrane, a conjugate pad, an absorbent pad, a polystyrene backing card, and a detection zone containing the test line (T) and control line (C). A NC membrane is attached to the backing card. One end of the NC membrane is connected to an absorbent pad, and the other end to a conjugate pad, which is overlapped with a sample pad. The NC membrane contains a control line (C) coated with biotin‐BSA and a parallel test line (T) coated with anti‐digoxigenin antibody. The conjugate pad is coated with streptavidin‐labeled fluorescent microspheres (Nanjing Dongna Biotechnology Co. Ltd., Nanjing, China). Hydrophilic glass fiber was selected as the sample pad and conjugate pad. Both pads were immersed in a mixed buffer solution (0.02 M phosphate buffer containing 1 wt% PEG, 1 wt% P300, 0.05 wt% casein, 0.05 wt% fetal bovine serum protein, and 0.01 wt% trehalose) and dried for subsequent use. The streptavidin‐labeled fluorescent microsphere (10 mg/mL) was sprayed onto the conjugate pad and then dried, yielding an immobilized conjugate pad. The anti‐digoxigenin antibody was diluted to 2 mg/mL with 0.02 M phosphate‐buffered saline (pH 7.4), and biotin‐BSA was diluted to 20 μg/mL with the same buffer. Both solutions were striped onto the NC membrane and then dried. The NC membrane was attached to the center of an adhesive PVC backing card. Then the immunochromatographic test strips were assembled and stored in the dark at 4°C.

### 2.3 Design of RPA Primers

Primers with a target fragment of 364 bp were designed based on the GenBank nucleotide sequence database (http://www.ncbi.nlm.nih.gov) for the specific target gene *mdh* of *Klebsiella pneumoniae* . Upstream primer F: 5′‐GCGTGGCGGTAGATCTAAGTCATA‐3′; Downstream primer R: 5′‐TTCAGCTCCGCCACAAAGGTA‐3′. The primers were synthesized by Shenggong Biotechnology (Shanghai) Co. Ltd. Label the 5′ end of upstream primer F with biotin and the 5′ end of downstream primer R with digoxigenin.

### 2.4 Establish RPA Technology

RPA reaction system: upstream and downstream primers (10 pmol/μL) 2.4 μL, Rehydration Buffer 29.4 μL, *Klebsiella pneumoniae* DNA 2 μL, ddH_2_O 11.2 μL, vibration, centrifugation; Finally, add 2.5 μL of 280 mmol/L MgAc, oscillating, centrifugal; After incubation at a constant temperature of 39°C for 30 min, extract the amplified product with phenol/chloroform, and then take 5 μL of the purified product and mix with the loading buffer in proportion. After loading it into the agarose gel with a concentration of 1%. The electrophoresis analysis is carried out under the condition of 140 V, 30 min, and observed and recorded under ultraviolet.

### 2.5 Purification of Products

Add 5 μL RPA amplification products and 9 μL Si‐MNP (Nanjing Dongna Biotechnology Co. Ltd., Nanjing, China) well and let them stand at room temperature for 5 min to fully adsorb DNA fragments by magnetic beads. Then fix the SiMNPs‐DNA complex with a magnet for 2 min, remove the waste liquid, wash it with 85% alcohol solution for 2 min, repeat twice, and let it stand for 4 min. Finally, remove the magnet, add 100 μL of elution buffer, shake well, let it stand for 5 min, elute the DNA from the magnetic beads, and then use a magnet to adsorb the magnetic beads in the tube. The supernatant is the purified DNA. The purified RPA product can be used for FICTS detection or stored at −20°C. The entire process is shown in Figure 2.

**FIGURE 2:**
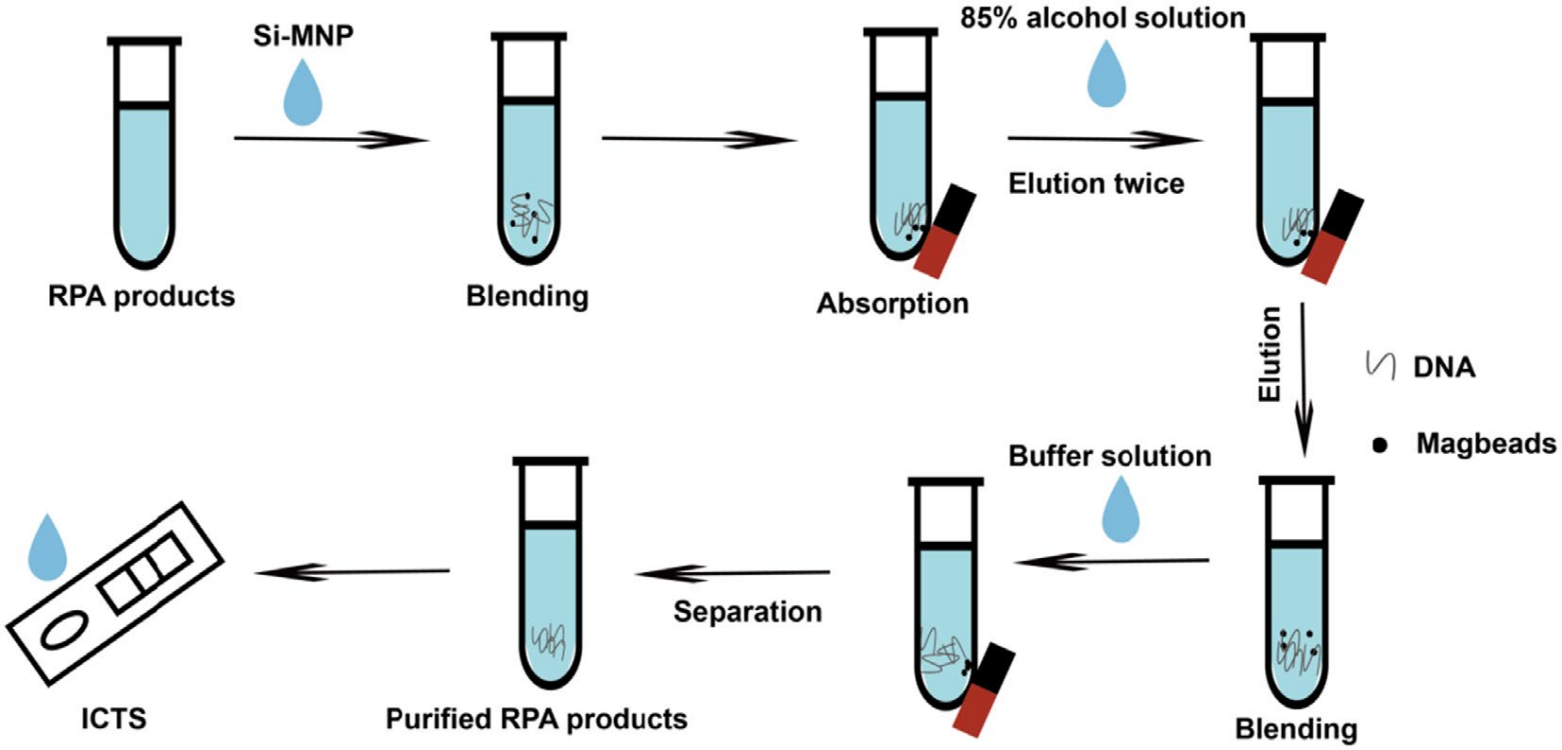
Schematic diagram of RPA products purified by Si‐MNP.

### 2.6 Development of RPA‐FICTS Detection System for KP

Add 100 μL purified RPA product directly to the FICTS sample pad at the vertical position indicated on the test paper as the specific test line capture site; meanwhile, add 100 μL PBS solution as blank control. After 2 min, read fluorescence values using the Nanoeasy 1700 ICTS sensor (Nanoeast, Nanjing, China). The whole process of quantifying RPA products takes less than 3 min.

### 2.7 Sensitivity and Specificity of RPA‐FICTS Detection

The specificity of RPA‐FICTS detection was evaluated using the standard strain of *Klebsiella pneumoniae* (ATCC 700603), 19 clinical isolates of KP, and 7 reference strains (nKP). The RPA amplification is carried out using 9.03 × 10 to 9.03 × 10^−7^ ng/μL standard DNA concentrations to assess the minimum detection limit of RPA‐GE and RPA‐FICTS. At the same time, using ddH_2_O instead of DNA as a negative control, and measuring each concentration three times by FICTS sensors. For fluorescence threshold detection, the detection limit signal value is defined as 500. Positive result signal value is greater than 500 and negative result signal value is less than 500.

### 2.8 Quantitative RTFQ‐PCR Detection

RTFQ‐PCR reactions were performed on a LightCycler480 II fluorescent quantitative PCR system (Roche, Rotkreuz, Switzerland) using 2× SG Fast qPCR Master Mix (B639271, BBI, Roche). Each 10 μL reaction mixture includes 5 μL 2× SybrGreen qPCR master mix, 0.2 μL primer‐F (10 μmol/L), 0.2 μL primer‐R (10 μmol/L), 1 μL DNA template and up to 10 μL sterile deionized water. Cyclical conditions are as follows: initial variability at 95°C for 3 min, at 95°C, 15 s, 60°C, 30 s, 45 cycles, instead of encoding according to the instrument dissociation guide. Then, use different concentrations of *Klebsiella pneumoniae* gDNA (4.34 × 10^7^–4.34 × 10^3^copies/μL) to detect the sensitivity of RTFQ‐PCR (*mdh*). The experiment was repeated three times for both the negative control group and the positive control group.

## 3 Results

### 3.1 The Principle of FICTS Quantitative Testing Platform Based on the Nanofluorescent Microspheres (nFM)

Genomic DNA was extracted, and the specific primers of genus‐specific gene *mdh* (F and R) were specifically designed, synthesized, and labeled with biotin and digoxigenin, respectively. Subsequently, RPA isothermal amplification was performed. The RPA product was purified to remove unwanted byproducts of the amplification reaction, such as preventing competitive inhibition and reducing background noise. Then the reaction product is vertically dropped onto an FICTS immunochromatographic strip based on the nanofluorescent microspheres nFM. One end of the product is labeled with biotin and bound to FM‐labeled streptavidin on FICTS, while the other end is labeled with digoxigenin and binds to the anti‐digoxigenin antibody in test line (T), redundant streptavidin‐coated nFM can be combined with biotin‐labeled BSA on the control line (C), thereby a portable FICTS sensor reader can detect and quantify the strong fluorescence signals. When the target DNA is present, a positive result shows two data points at the T‐line and C‐line positions on the test paper, while a negative result did not produce any result on the T‐line and shows a very low value (value < 500). The RPA‐FICTS method can therefore be used for rapid and specific analysis of genomic DNA isolated from KP. There were no false positives or false negatives in the experiment, and the results were comparable to the gold standard detection technique of RTFQ‐PCR (As shown in Figure 3).

**FIGURE 3:**
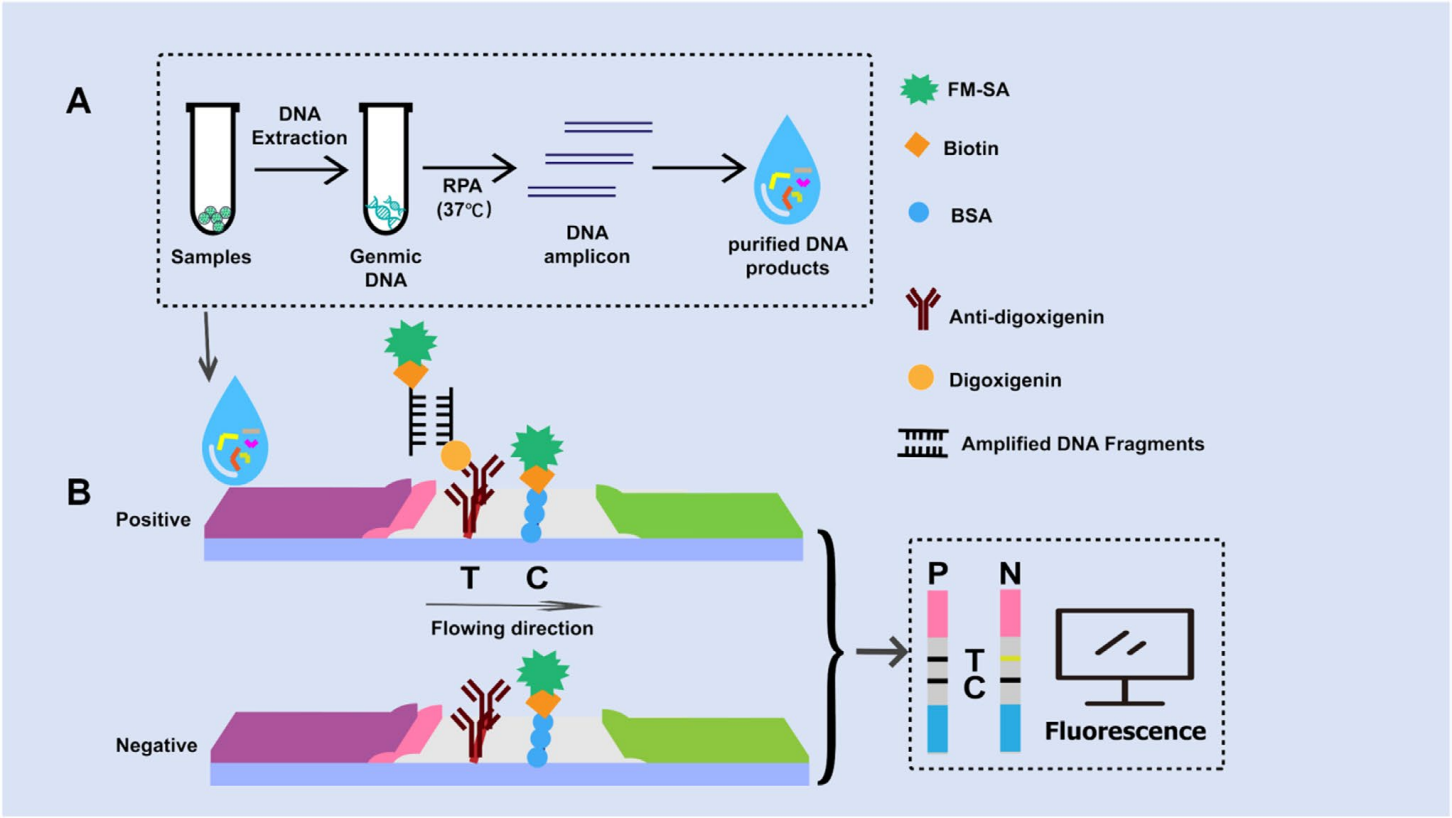
Schematic diagram of RPA‐FICTS method. (A) Schematic representation of an immunochromatographic test strip used for the testing of KP. (B) Photographs of packaged FICTS and internal structures, including sample pads, conjugate pads, detection areas with detection and control lines, and absorbent pad.

### 3.2 nFM‐SA Characteristics

Fluorescent nanofluorescent microspheres streptavidin (nFM‐SA) conjugates were prepared by the coupling method and characterised. Figure 4 indicates that the nFM before and after the SA modification exhibit a standard spherical morphology. Transmission electron microscopy (TEM) images show that compared to free nFM, the surface of nFM‐SA beads is covered with a membrane‐like structure. Experimental results prove that the nFM‐SA has the characteristics of high fluorescence intensity, good dispersion, narrow particle size distribution as well as stable performance.

**FIGURE 4:**
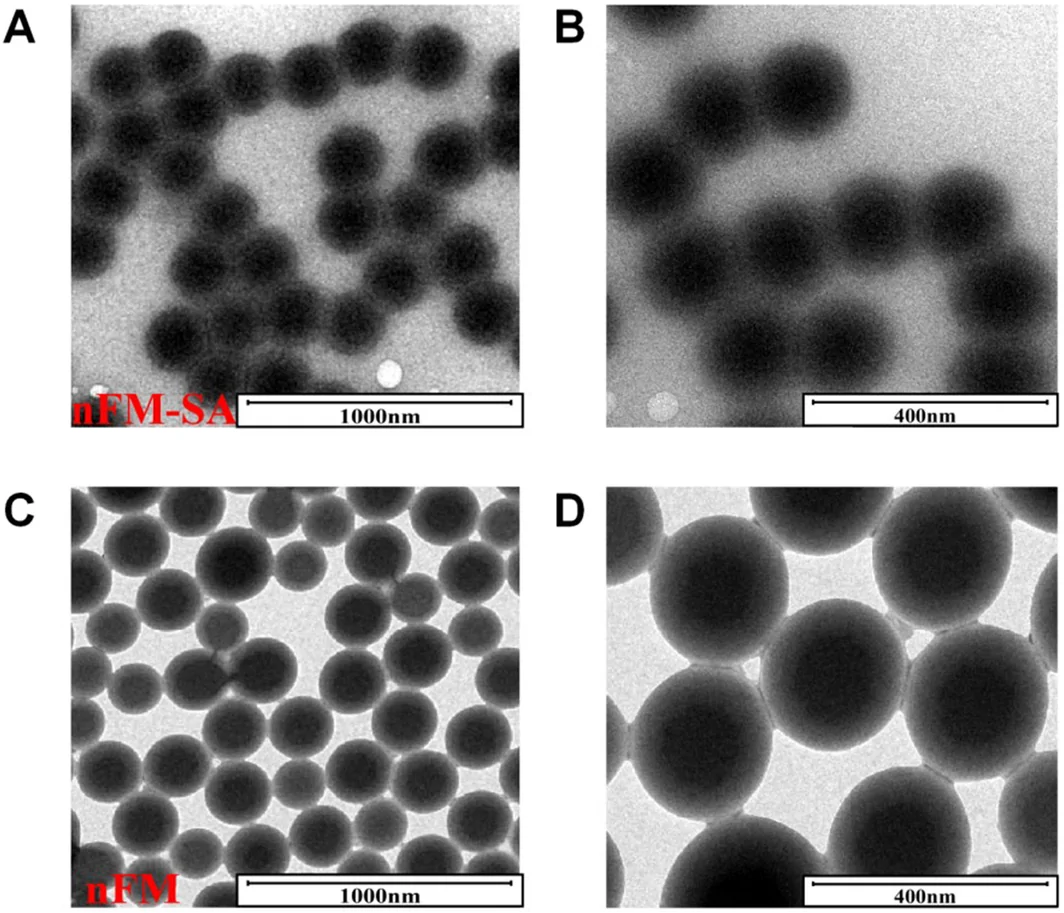
Characterization of nFM and nFM‐SA conjugates. (A and B) TEM images of synthesized nFM‐SA conjugates; (C and D) TEM images of synthesized nFM conjugates.

### 3.3 Verify the Feasibility of RPA‐FICTS Platform for Nucleic Acid Detection

To verify the feasibility of the RPA‐FICTS platform for nucleic acid detection, we selected the *Klebsiella* *pneumoniae*‐specific gene *mdh* for testing. The possibility of nonspecific amplification was minimized by visualizing the RPA product by agarose gel electrophoresis (GE) under UV light and confirming product size and amplification by comparison with DNA markers (TaKaRa, Dalian, China). The results showed that the RPA product was successfully replicated, while the negative control group did not yield replication results (Figure 5).

**FIGURE 5:**
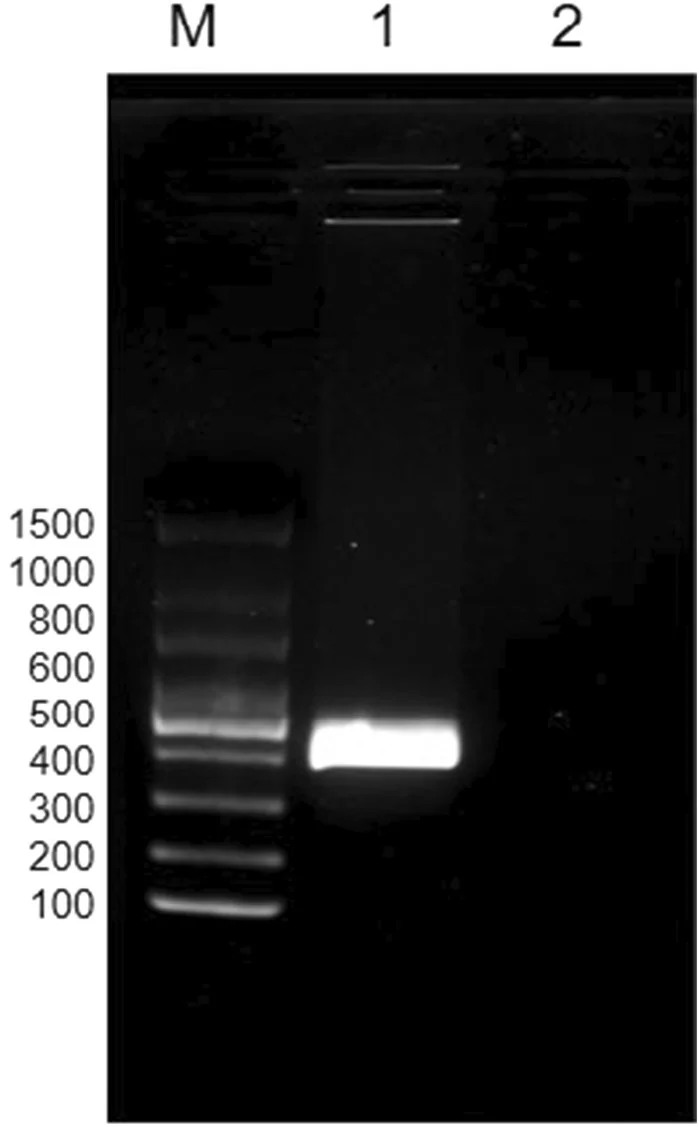
Establishment and finalization of RPA.

### 3.4 Sensitivity of RPA‐FICTS Detection Platform

Sensitivity tests show the lower detection limit of the method. The RPA‐FICTS method is used to detect the genus specific gene *mdh* of KP, with a concentration of 9.03 × 10 ng/μL and continuously dilute 10 times. In RPA‐FICTS, the range of fluorescence signal values after standard DNA dilution is 53,891 to 321.6, and the *mdh* concentration ranges from 9.03 × 10 ng/μL reduced to 9.03 × 10^−9^ ng/μL. The relationship between different concentrations and detection values is {*y* = 49,187.49/1 + e^(−2.381–1.02*x*)^}, and correlation analysis showed that the Pearson's correlation coefficient (Pearson's *r*) between DNA concentration and FICTS efficiency was *R* ^2^ = 0.97 (Figure 6). The results showed that the sensitivity of RPA‐FICTS to detect *mdh* was 9.03 × 10^−8^ ng/μL.

**FIGURE 6:**
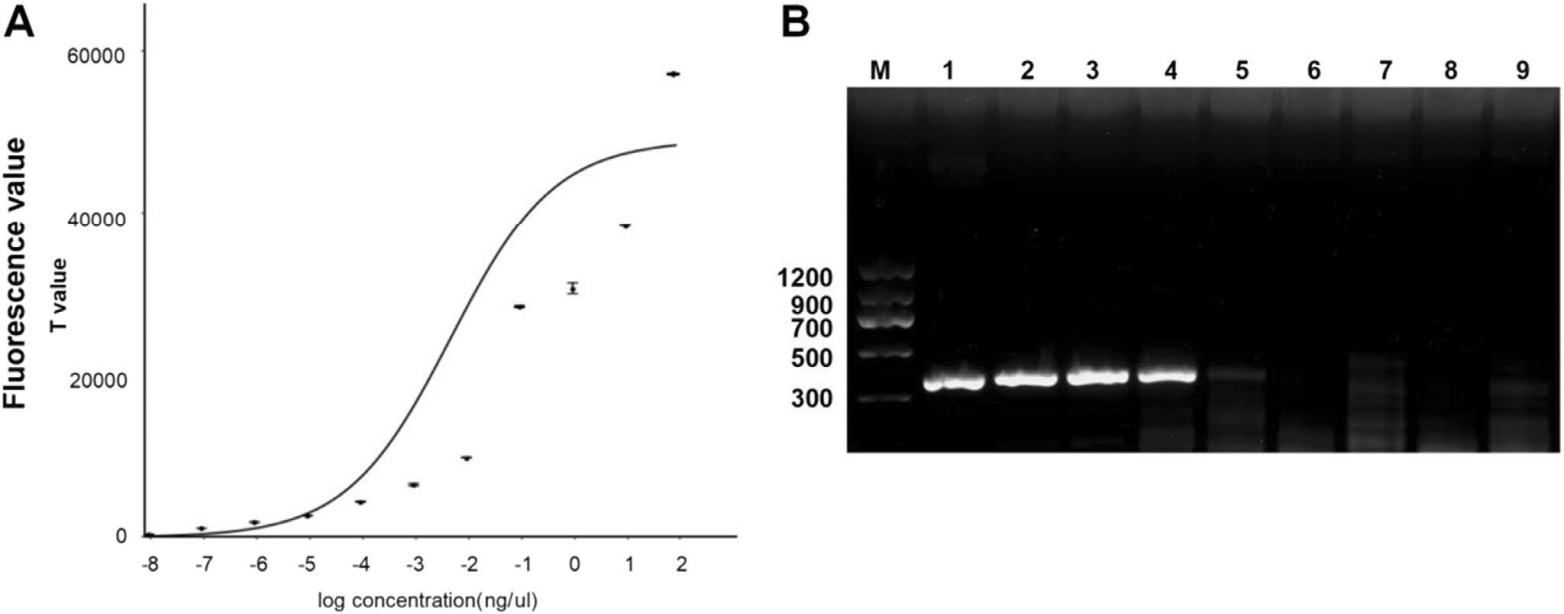
Sensitivity of RPA‐FICTS for detecting KP. (A) Correlation curves are plotted with log DNA concentration values on the abscissa and FICTS signal values (*T*‐values) on the ordinate. Each value was obtained from three independent scans, with error bars representing the standard deviation. (B) Sensitivity of RPA‐GE detection using serially diluted *Klebsiella pneumoniae* (*mdh*): Lane M, DL1500 marker; lanes 1–8, DNA concentration from 9.03 × 10^1^ to 9.03 × 10^−6^ ng/μL; lanes 9, negative control. The size of the RPA products (*mdh*) was 364 bp.

### 3.5 The Specificity of RPA‐FICTS Detection Platform and Its Detection of Clinical Specimens

To validate the specificity of the RPA‐FICTS detection platform, 19 clinical samples of *Klebsiella pneumoniae* biological pathogens and 7 non‐ *Klebsiella pneumoniae* biological pathogens in infants were tested. As a result, a characteristic amplified band emerged from the standard *Klebsiella pneumoniae* strain (ATCC 700603) and 19 clinical samples, while neither the non‐*Klebsiella pneumoniae* strain nor the negative control showed any amplification bands (Figure 7). RPA‐FICTS was performed on 19 cases of *Klebsiella pneumoniae* and 7 cases of non‐*Klebsiella pneumoniae* in infants and young children. The results indicate that FICTS technology has high specificity for detecting KP, showing no cross‐reactivity with nKP, which is consistent with traditional detection methods (Table 2).

**FIGURE 7:**
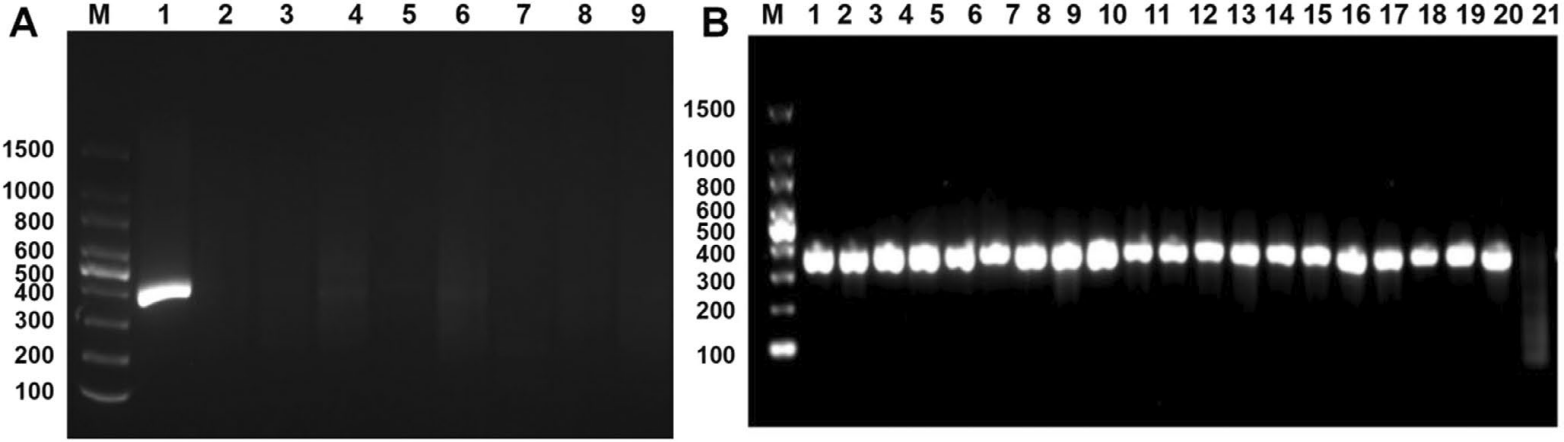
Specificity of RPA‐GE for detection of KP and 19 clinical samples. (A) Specificity of RPA‐GE detection using a standard KP strain and 7 nKP cases: Lane M, DL 1500 marker; lanes 1, the standard strain of KP; lanes 2–8, nKP; lanes 9, negative control. The size of the RPA products (*mdh*) was 364 bp. (B) RPA‐GE was used to detect KP of the standard strain and 19 clinical samples: Lane M, DL 1500 marker; lanes 1, the standard strain of KP; lanes 2–20, 19 clinical specimens of KP in infants; lanes 21, negative control. The size of the RPA products (*mdh*) was 364 bp.

**TABLE 2:** RPA‐FICTS specificity results.

No.	Sample type and species	RPA‐GE	RPA‐FICTS	Vitek 2 compact
1	*Klebsiella pneumoniae* (ATCC 700603)	+	+	+
2	*Klebsiella pneumoniae* (A13)	+	+	+
3	*Klebsiella pneumoniae* (A19)	+	+	+
4	*Klebsiella pneumoniae* (A20)	+	+	+
5	*Klebsiella pneumoniae* (A22)	+	+	+
6	*Klebsiella pneumoniae* (A23)	+	+	+
7	*Klebsiella pneumoniae* (A25)	+	+	+
8	*Klebsiella pneumoniae* (A28)	+	+	+
9	*Klebsiella pneumoniae* (A34)	+	+	+
10	*Klebsiella pneumoniae* (A35)	+	+	+
11	*Klebsiella pneumoniae* (A36)	+	+	+
12	*Klebsiella pneumoniae* (A40)	+	+	+
13	*Klebsiella pneumoniae* (A49)	+	+	+
14	*Klebsiella pneumoniae* (A57)	+	+	+
15	*Klebsiella pneumoniae* (A59)	+	+	+
16	*Klebsiella pneumoniae* (A61)	+	+	+
17	*Klebsiella pneumoniae* (A63)	+	+	+
18	*Klebsiella pneumoniae* (A65)	+	+	+
19	*Klebsiella pneumoniae* (A66)	+	+	+
20	*Klebsiella pneumoniae* (A125)	+	+	+
21	*Staphylococcus aureus*	−	−	−
22	*Shigella flexneri*	−	−	−
23	*Acinetobacter baumannii*	−	−	−
24	*Maltodomonas maltophilia*	−	−	−
25	*Staphylococcus epidermidis*	−	−	−
26	*E. coli*	−	−	−
27	*Salmonella*	−	−	−
28	Negative control	−	−	−

### 3.6 RTFQ‐PCR Quantitative Detection

As shown in Figure 8A, the logarithm of DNA concentration and the RTFQ‐PCR cycle threshold (Ct) have a good linear relationship. The linear regression equation is *y* = −3.206*x* + 34.19, and the *R* ^2^ value is greater than 0.99. The minimum detection limit for plasmids containing the target gene is 67.3 copies/μL, which corresponds to a DNA concentration of 2.5 × 10^−6^ ng/μL. Figure 8B demonstrates the high reproducibility of the results of the detection of different concentrations of KP DNA. The selected *mdh* genes and specific primers can isolate KP from other bacteria and are very repetitive and sensitive. It shows that the sensitivity of FICTS is essentially the same as RTFQ‐PCR, but the cost of RPA‐FICTS is lower and the results of FICTS are easier to interpret. Parallel detection of clinical samples was conducted using RTFQ‐PCR and RPA‐FICTS, and one representative specimen produced matching positive signals with *C*_*t*_ = 14.0855 of RTFQ‐PCR and a fluorescence reading of 32,213 of RPA‐FICTS, respectively.

**FIGURE 8:**
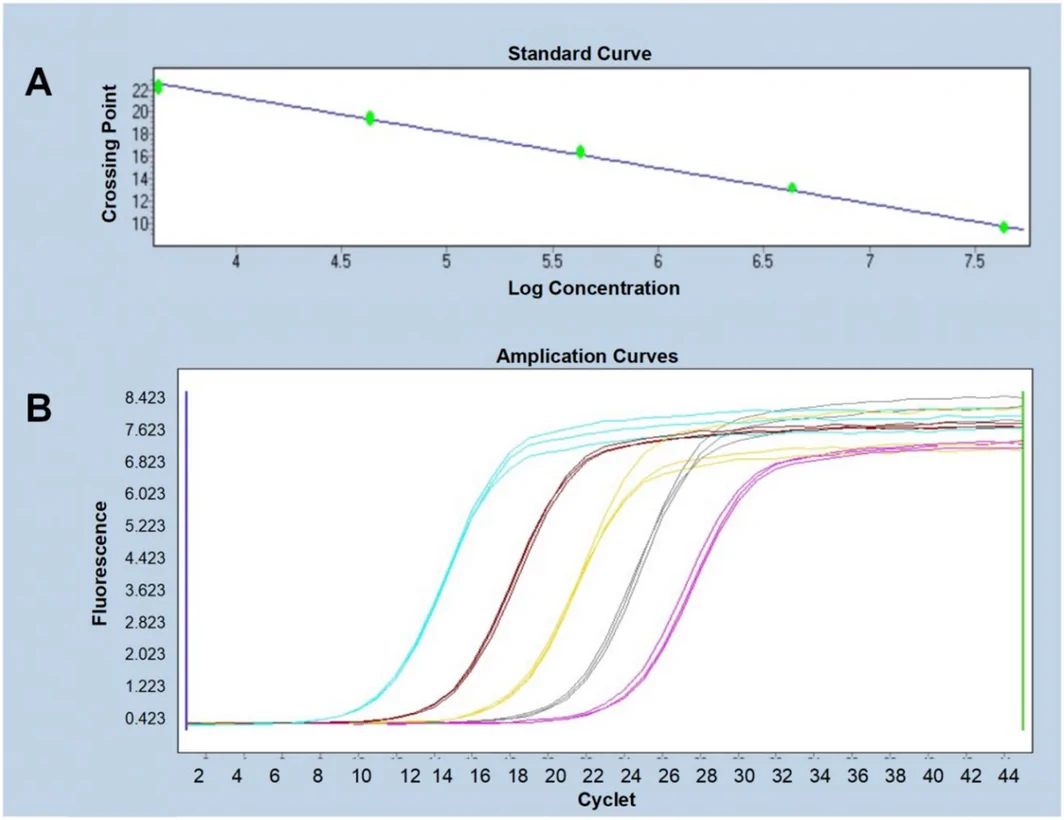
(A) Standard RTFQ‐PCR curve for the detection of KP from 1.43 × 10^−2^ to 1.43 × 10^−6^ ng/μL of DNA samples; (B) RTFQ‐PCR amplification curves at various concentrations. The numbers 1–5 denote various concentrations.

## 4 Discussion

Research has found that *Klebsiella pneumoniae* is an important cause of neonatal bacteremia and death. Due to increasing antibiotic resistance and the emergence of highly virulent strains, *Klebsiella pneumoniae* infection has become increasingly difficult to treat [21, 22, 23].

RPA technology is a novel isothermal amplification technique that utilizes recombinant enzymes, strand displacement polymerase, and single stranded binding proteins. This is one of the nucleic acid amplification methods used in molecular diagnostics. Unlike conventional nucleic acid separation and detection methods, which take a minimum of 3 h, this system can complete the entire process in 1 h [24, 25, 26]. Meanwhile, its reaction does not require expensive PCR amplification equipment, which has the advantages of simple operation, fast reaction speed, low equipment requirements, and mild reaction conditions [27]. Most test strips are only used for qualitative analysis, but in the assays, biotin‐labeled amplified products were obtained using streptavidin‐modified nFM sprayed onto the test strip. Compared to colloidal gold, nFM has fluorescence signal intensity, wide linear range, good throughput and repeatability, high sensitivity and accurate quantitative detection [28]. In this study, we combine RPA technology with FICTS technology to establish a new RPA‐FICTS detection platform. Research has found that the platform can detect as low as 9.03 × 10^−8^ ng/μL of *Klebsiella* *pneumoniae*; meanwhile, its specificity is high, 19 clinical *Klebsiella pneumoniae* samples can be detected, and its detection sensitivity is 5 orders of magnitude higher than that of traditional agarose gel electrophoresis. At the same time, it overcomes the shortcomings of traditional agarose gel electrophoresis, such as low resolution and strong carcinogenicity, which can cause great harm to the experimenter and the surrounding environment [29, 30].

In recent years, numerous detection methods for *K. pneumoniae* have been developed. Through comparison, we found that conventional microbiological methods are time‐ and labor‐intensive, typically requiring 2–3 days to obtain reliable results. PCR‐GE is relatively slow (220 min), has low sensitivity, uses toxic nucleic acid stains, and does not provide quantitative results; PCR‐ICTS [2] is easy to use, precise and specific, the false positive rate is very low and the detection speed increases to 160 min; RTFQ‐PCR has a fast detection time (160 min) and high detection sensitivity. Moreover, this detection method requires skilled technicians and a large amount of specialised equipment, making it very expensive and limited. In contrast, the RPA‐FICTS platform developed in this study has a much shorter detection time (55 min), requires no expensive RTFQ‐PCR instrument, and has low detection and labor costs. The results of a comparison of different methods for detecting *Klebsiella pneumoniae* are presented in Table 3.

**TABLE 3:** Comparison of detection method parameters for KP.

Method	Sensitivity	Demands for instrumentation	Potential for specific DNA sequence	Inspection cost	Times
Traditional microbiological method	Low	Medium	No	Medium	2–3 days
PCR‐GE	Medium	Medium	No	Medium	220 min^a^
PCR‐ICTS	High	Medium	Yes	Low	160 min^b^
RTFQ‐PCR	High	High	Yes	High	160 min^c^
RPA‐FICTS^e^	High	Low	No	Low	55 min^d^

^a^ 220 min include the time of DNA extraction, PCR, and electrophoresis procedure.

^b^ 160 min include the time of DNA extraction, PCR, purification, and ICTS procedure.

^c^ 160 min include the time of DNA extraction and RTFQ‐PCR procedure.

^d^ 55 min include the time of DNA extraction, RPA, purification and ICTS procedure.

^e^ The new detection method of RPA‐FICTS developed in our study.

Therefore, the RPA‐FICTS quantitative detection platform developed in this study has the characteristics of high sensitivity, high specificity, easy operation, low cost, and high detectability. It is a practical tool for quantitatively and accurately determining the presence or absence of *Klebsiella pneumoniae* infection in patients, especially infants, and provides a new method for routine clinical detection of *Klebsiella pneumoniae* . Taking the single‐target detection platform established in this study as the foundation, we will further develop a multiplex panel for respiratory pathogens that includes *Streptococcus pneumoniae* and other pathogenic microorganisms. This panel enables simultaneous detection of multiple typical respiratory pathogens in clinical specimens and provides new strategies to improve the differential diagnosis efficiency of respiratory tract infections.

## 5 Conclusion

This article introduces a quantitative detection platform of RPA‐FICTS which can test *Klebsiella pneumoniae* in infants as low as 9.03 × 10^−8^ ng/μL. The results indicate that the new RPA‐FICTS technology offers simple operation, rapid detection, and low cost, without the need for expensive detection instruments. At the same time, it has strong specificity and high sensitivity, making it easy to quickly detect *Klebsiella pneumoniae* in infants, providing a new method for traditional clinical microbiological detection.

## Author Contributions

Conception and design: Ying Chen, Li Wang, Xin Wang. Administrative support: Ying Chen, Li Wang, Hanzhong Yu. Provision of study materials: Yi Lu, Xin Wang. Collection and assembly of data: Yiping Liu, Yumeng Lu, Feifan Zhu, Xing Lv. Data analysis and interpretation: Yiping Liu, Ying Chen, Minghui Chen, Yi Lu. Manuscript writing: All authors. Final approval of manuscript: All authors.

## Funding

This work was sponsored by the Jiangsu Provincial Natural Science Foundation (BK20231184), Innovation and Entrepreneurship Projects of Xuzhou Medical University Science Park (CXCYZX2024005), Jiangsu Province Vice President of Science and Technology Project (FZ20210142), National College Student Innovation Training Program (202210313033Z, 202410313028Z), the Key Scientific Research Project of Jiangsu Provincial Health Commission (ZD2022050), and the Xuzhou “Health Peak” Team Training Project (2025DF01).

## Ethics Statement

This study was approved by the Medical Ethics Committee of the Affiliated Hospital of Xuzhou Medical University (Approval No. XYFY2024‐KL162‐01) in accordance with the principles of the Declaration of Helsinki. Informed consent was obtained from all individual participants included in the study.

## Conflicts of Interest

The authors declare no conflicts of interest.

## Data Availability

All data generated or analyzed during this study that support the findings are included in the published article. Additional raw data, including the detailed sample source information, are not publicly available due to ethical restrictions. Requests to access the data should be directed to the corresponding author, Ying Chen, at chenyingphd@163.com.
